# The failure of four bootstrap procedures for estimating confidence intervals for predicted-to-expected ratios for hospital profiling

**DOI:** 10.1186/s12874-022-01739-x

**Published:** 2022-10-14

**Authors:** Peter C. Austin

**Affiliations:** 1 ICES, G106, 2075 Bayview Avenue, Toronto, ON M4N 3M5 Canada; 2 Institute of Health Management, Policy and Evaluation, University of Toronto, Toronto, ON Canada; 3 Sunnybrook Research Institute, Toronto, ON Canada

**Keywords:** Hospital profiling, Hospital report cards, Random effects models, Multilevel analysis

## Abstract

**Background:**

Healthcare provider profiling involves the comparison of outcomes between patients cared for by different healthcare providers. An important component of provider profiling is risk-adjustment so that providers that care for sicker patients are not unfairly penalized. One method for provider profiling entails using random effects logistic regression models to compute provider-specific predicted-to-expected ratios. These ratios compare the predicted number of deaths at a given provider given the case-mix of its patients with the expected number of deaths had those patients been treated at an average provider. Despite the utility of this metric in provider profiling, methods have not been described to estimate confidence intervals for these ratios. The objective of the current study was to evaluate the performance of four bootstrap procedures for estimating 95% confidence intervals for predicted-to-expected ratios.

**Methods:**

We used Monte Carlo simulations to evaluate four bootstrap procedures: the naïve bootstrap, a within cluster-bootstrap, the parametric multilevel bootstrap, and a novel cluster-specific parametric bootstrap. The parameters of the data-generating process were informed by empirical analyses of patients hospitalized with acute myocardial infarction. Three factors were varied in the simulations: the number of subjects per cluster, the intraclass correlation coefficient for the binary outcome, and the prevalence of the outcome. We examined coverage rates of both normal-theory bootstrap confidence intervals and bootstrap percentile intervals.

**Results:**

In general, all four bootstrap procedures resulted in inaccurate estimates of the standard error of cluster-specific predicted-to-expected ratios. Similarly, all four bootstrap procedures resulted in 95% confidence intervals whose empirical coverage rates were different from the advertised rate. In many scenarios the empirical coverage rates were substantially lower than the advertised rate.

**Conclusion:**

Existing bootstrap procedures should not be used to compute confidence intervals for predicted-to-expected ratios when conducting provider profiling.

## Background

Provider profiling involves the comparison of outcomes between healthcare providers [1]. Examples of provider profiling include comparisons of outcomes between hospitals following coronary artery bypass graft (CABG) surgery and following hospitalization for acute myocardial infarction (AMI) [2–8]. An important component of provider profiling is risk-adjustment, so that providers that care for sicker patients are not unfairly penalized [1].

Historically, the most common approach to risk-adjustment was to compute provider-specific observed-to-expected ratios, comparing the observed mortality at each provider to the mortality that would be expected given the case-mix of its patients. An observed-to-expected ratio can be computed by using a conventional logistic regression to regress the binary outcome (e.g., death within 30 days of the CABG surgery or of hospital admission for AMI). Using the fitted model, the predicted probability of the outcome, conditional on their baseline covariates, is determined for each patient. These probabilities are summed up within each provider to generate the expected number of deaths at each provider given the case-mix of its patients. Then the observed number of deaths is divided by the expected number of deaths, to produce the provider’s observed-to-expected ratio (this ratio can be multiplied by the overall sample-wide event rate to produce a risk-adjusted mortality rate). Providers whose ratio is greater than one have observed mortality that exceeds the mortality that would be expected given the case-mix of its patients. Providers whose ratio is less than one have observed mortality that is less than the mortality that would be expected given the case-mix of its patients. Hosmer and Lemeshow provided a closed-form expression for the standard error of the observed-to-expected ratio, allowing for estimation of confidence intervals around the ratio [9]. Providers whose estimated confidence interval excludes the null value of one can be classified as having outcomes that are significantly different from expected. In addition to providing a closed-form expression for the standard error of the observed-to-expected ratio, Hosmer and Lemeshow suggested that the bootstrap could be used to construct confidence intervals for the provider-specific observed-to-expected ratios. While an empirical comparison of bootstrap confidence intervals with those derived using asymptotic methods was conducted in a single dataset, the performance of these intervals was not evaluated using simulations. Indeed, the authors suggested that “a detailed simulation study is needed before we can recommend a definitive choice between methods”.

Krumholz and colleagues suggested a modification of the observed-to-expected ratio [10]. Rather than use a conventional logistic regression model, the binary outcome is regressed on baseline characteristics using a random effects logistic regression model that incorporates provider-specific random effects:

1 $${\text{logit}}(p_{ij} = \Pr (Y_{ij} = 1)) = \beta_{0} + \beta_{0j} + {\varvec{\beta X}}_{ij}$$

where $p_{ij}$ denotes the probability of death for the *i*th patient at the *j*th provider (Y_ij_ = 1 dead/Y_ij_ = 0 alive) and where $\beta_{0j} \sim N(0,\tau^{2} )$ are the provider-specific random effects. The observed-to-expected ratio is modified by replacing the observed number of deaths by the predicted number of deaths given the case-mix of the provider’s patients. For each patient, the probability of death is $\frac{{\exp (\hat{\beta }_{0} + \hat{\beta }_{0j} + {\varvec{\hat{\beta }X}}_{ij} )}}{{1 + \exp (\hat{\beta }_{0} + \hat{\beta }_{0j} + {\varvec{\hat{\beta }X}}_{ij} )}}$. These probabilities are summed up within each provider to obtain the predicted number of deaths for that provider given the case-mix of its patients. For each patient, the probability of death if he or she were treated at an average provider is $\frac{{\exp (\hat{\beta }_{0} + {\varvec{\hat{\beta }X}}_{ij} )}}{{1 + \exp (\hat{\beta }_{0} + {\varvec{\hat{\beta }X}}_{ij} )}}$ (note that this differs from the previous expression only in the removal of the predicted cluster-specific random effect $\hat{\beta }_{0j}$). These probabilities are summed up within each provider to obtain the expected number of deaths had those patients been treated at an average provider. The ratio of these two quantities is the predicted-to-expected ratio and is used as a measure of provider performance. It has an interpretation similar to that of the observed-to-expected ratio. Krumholz and colleagues argue that an advantage of this approach is that the use of a random effects model explicitly accounts for the within-provider correlation in outcomes and that the model therefore explicitly accounts for underlying quality differences between providers. Furthermore, the use of the predicted, rather than the expected, number of deaths makes it simpler to include providers with a small number of patients. When outcomes are rare, a low-volume provider may have zero observed outcomes, despite having a predicted number of outcomes that is greater than zero. Despite the attractive features of the use of predicted-to-expected ratios, a closed-form variance estimator for the ratio has not been developed. Furthermore, the performance of the bootstrap for estimating confidence intervals for these ratios has not been systematically examined.

The objective of this study was to evaluate the performance of different bootstrap estimators for provider-specific predicted-to-expected ratios. We consider the conventional bootstrap procedure for non-clustered data, a bootstrap procedure for multilevel data, and a recently-proposed parametric bootstrap procedure for estimating confidence intervals for predicted cluster-specific random effects [11]. The paper is structured as follows: in Bootstrap procedures for predicted-to-expected ratios, we describe different candidate bootstrap procedures for estimating confidence intervals for predicted-to-expected ratios. In Monte Carlo simulations: Methods, we describe the design of a series of Monte Carlo simulations to evaluate the performance of different bootstrap procedures. The results of these simulations are summarized in Monte Carlo simulations: Results. In Case study, we provide a case study illustrating the application of these methods to a sample of patients hospitalized with AMI. Finally, we summarize our conclusions and place them in the context of the literature in Discussion.

## Bootstrap procedures for predicted-to-expected ratios

In this section we provide a brief review of bootstrap procedures for clustered (or multilevel) data and a brief commentary on why some methods are not appropriate for making inferences about cluster-specific predicted-to-expected ratios.

### The simple or naïve bootstrap

The conventional bootstrap draws a random sample with replacement from the original sample, such that the random sample has the same size as the original sample [12]. While the original bootstrap procedure is not recommended for clustered data, we include it here as it is the basis of the subsequent bootstrap procedures.

### Multilevel bootstrap procedures

Three different bootstrap procedures for use with linear mixed models have been described by van der Leeden and colleagues, by Goldstein, and by Carpenter and colleagues: the parametric bootstrap, the residuals bootstrap, and the non-parametric bootstrap [13–16]. We will describe these in the context of the random effects logistic regression described in formula (1). We assume that there are J clusters.

The parametric bootstrap estimates the random effects logistic regression model in [1]. In particular, one obtains an estimate, $\hat{\tau }^{2}$, of the variance of the cluster-specific random effects. Then, for each of the J clusters, one draws a cluster-specific effect from this distribution: $\beta_{0j}^{{{\text{bs}}}} \sim N(0,\hat{\tau }^{2} ),\;j = 1,...,{\text{J}}$. One then determines the probability of the outcome occurring for each subject as: ${\text{logit}}(p_{ij}^{{{\text{bs}}}} ) = \hat{\beta }_{0} + \beta_{0j}^{{{\text{bs}}}} + {\varvec{\hat{\beta }X}}_{ij}$. A new binary outcome $Y_{ij}^{{{\text{bs}}}}$ is simulated from a Bernoulli distribution with subject-specific parameter $p_{ij}^{{{\text{bs}}}}$. A random effects logistic regression model is then fit to the data $(Y_{ij}^{{{\text{bs}}}} ,{\mathbf{X}}_{ij} )$. The predicted-to-expected ratio is then computed for each hospital using the fitted model. This process constitutes one bootstrap iteration.

The residuals bootstrap is very similar to the parametric bootstrap described above. It differs from the parametric bootstrap in that, rather than simulating cluster-specific effects from the estimated distribution $N(0,\hat{\tau }^{2} )$, one simulates the cluster-specific effects from their empirical distribution. The empirical distribution of predicted cluster-specific effects begins with $\{ \hat{\beta }_{0j} |j = 1,...,J\}$. These are then standardized to have mean zero and are inflated so that their sample variance is equal to $\hat{\tau }^{2}$.

The non-parametric bootstrap, also referred to as the cases bootstrap, takes a bootstrap sample of the clusters. Once a cluster has been selected, all that cluster’s subjects are included in the bootstrap sample. Note that an average bootstrap will contain 63.2% of the clusters and omit 36.8% of the clusters. Importantly, those clusters that are contained multiple times in a given bootstrap sample are given different cluster identifiers so that they are treated as distinct clusters.

As described elsewhere, these three bootstrap procedures allow one to make inferences about model parameters (e.g., regression coefficients and the variance of the random effects), however, they cannot be used to make inferences about the predicted cluster-specific random effects nor on quantities derived from them [11]. With both the parametric and residuals bootstrap procedures, for a given cluster, the mean of the simulated cluster-specific random effects will be zero across the bootstrap replicates. Accordingly, the mean simulated cluster-specific random effect will not be an acceptable estimator for the predicted cluster-specific random effect for that cluster. If, for a given cluster, the mean simulated cluster-specific random effect is zero, that implies that, on average, the predicted-to-expected ratio will have a central value of one. Thus, when constructing percentile-based bootstrap confidence intervals, the constructed intervals for all clusters will contain the null value. With the non-parametric or case bootstrap, a given cluster can be included multiple times in a given bootstrap sample. The different replicates of this cluster are given distinct cluster identifiers. When making inferences about the cluster-specific predicted random effects (and quantities derived from this such as the predicted-to-expected ratio), it is not clear which of these cluster replicates should be used. Furthermore, the consequences of omitting 36.8% of the clusters from a given bootstrap sample are unclear.

### Cluster-specific parametric bootstrap procedure based on predicted cluster-specific random effects

Austin and Leckie described a novel cluster-specific parametric bootstrap procedure for making inferences about cluster-specific random effects [11]. After estimating the random effects logistic regression model described by formula (1), one obtains the predicted cluster-specific random effects and estimates of their standard error: $\hat{\beta }_{0j}$ and ${\text{se}}(\hat{\beta }_{0j} )$, for *j* = 1,…,J. For each cluster, one then simulates a cluster-specific random effect: $\beta_{0j}^{{{\text{bs}}}} \sim N(\hat{\beta }_{0j} ,{\text{se}}(\hat{\beta }_{0j} )^{2} )$. Having simulated a cluster-specific random effect for each of the J clusters, one then inflates them (as with the residuals bootstrap) so that their sample variance is equal to $\hat{\tau }^{2}$. One then proceeds identically as with the parametric bootstrap or the residuals bootstrap. Note that this procedure differs from the parametric bootstrap procedure in that each cluster-specific random effect is drawn from its own distribution, rather than from the same distribution.

## Monte Carlo simulations: methods

We conducted a series of Monte Carlo simulations to examine the performance of different bootstrap procedures for estimating confidence intervals for hospital-specific predicted-to-expected ratios generated using random effects logistic regression models. The design of the simulations was informed by empirical analyses of patients hospitalized with AMI.

### Empirical analyses to inform the Monte Carlo simulations

We conducted a series of empirical analyses to determine the values of parameters that would be used in the data-generating processes in the subsequent Monte Carlo simulations. We used data from the Ontario Myocardial Infarction Database (OMID) which contains data on patients hospitalized with AMI in Ontario, Canada between 1992 and 2016 [17]. For the current study, we used data on 19,559 patients hospitalized with a diagnosis of AMI at 157 hospitals between April 1, 2016 and March 31, 2017. Hospital volumes of AMI patients ranged from 1 to 1,146, with a median of 52 (25^th^ and 75^th^ percentiles: 16 and 148, respectively).

We considered two binary outcome variables: death within 30 days of hospital admission and death within one year of hospital admission. Outcomes were determined through linkage with the provincial death registry. Of the 19,559 patients, 1479 (7.6%) died within 30 days of hospital admission, while 2951 (15.1%) died within one year of hospital admission.

We considered 11 variables for predicting mortality: age, sex, congestive heart failure, cerebrovascular disease, pulmonary edema, diabetes with complications, malignancies, chronic renal failure, acute renal failure, cardiogenic shock, and cardiac dysrhythmias. These 11 variables comprise the Ontario AMI mortality prediction model, which was derived in Ontario and was subsequently validated in Manitoba and California [18].

We used conventional logistic regression to regress each of the two binary outcomes (death within 30 days and within one year) on the 11 variables in the Ontario AMI mortality prediction model. For each of the two fitted models, we determined the linear predictor for each subject. Thus, each subject had two linear predictors: one for each of the two outcomes. Each of the two linear predictors was standardized to have mean zero and unit variance across the sample. Each binary outcome was then regressed on the standardized linear predictor using a random effects logistic regression model that incorporated hospital-specific random effects. We computed the residual intraclass correlation coefficient (ICC), which is equivalent to the variance partition coefficient (VPC), using the latent variable approach [19].

The mean intercept and the fixed slope for the random effects logistic regression model for 30-day mortality were -3.06 and 1.17, respectively, while the mean intercept and the fixed slope for the 1-year mortality model were -2.26 and 1.39, respectively. The residual ICC was 0.01 for both models, indicating that 1% of the variation in mortality unexplained by the standardized linear predictor was due to between-hospital differences. These quantities will be used in our subsequent data-generating processes.

### Factors in the Monte Carlo simulations

We allowed three factors to vary in our simulations: N (the number of patients per hospital), ICC (the intraclass correlation coefficient denoting the within-cluster homogeneity in the binary outcome), and the intercept and slope of the logistic regression model (which determine the prevalence of the binary outcome). N took on two values: 50 or 100 patients per hospital. ICC took on three values: 0.01, 0.02, and 0.05. The intercept and slope took on three combinations: (-3.06,1.17), (-2.26,1.39), and (0,1.39). The first intercept and slope were from the empirical analysis of 30-day mortality above. The second intercept and slope were from the empirical analysis of 1-year mortality above. The intercept in the third pair was set to zero so that the prevalence of the outcome would be approximately 0.5, allowing us to examine the performance of the bootstrap procedures in a scenario in which the prevalence of the outcome was high (the slope in the third pair was simply the slope from the 1-year mortality model). We used a full factorial design and thus considered 18 (2 × 3 × 3) scenarios.

### Data-generating process for clustered hospital data

We simulated data for subjects hospitalized at 50 hospitals (this quantity was fixed across all scenarios for computational reasons; increasing the number of clusters would have resulted in simulations that were too computationally intensive). Our objective was to examine coverage of estimated confidence intervals for hospital-specific predicted-to-expected ratios. Thus, it is important that these hospital-specific ratios be treated as fixed parameters that are fixed across simulation replicates. Thus, within each of the 18 different scenarios we generated 50 hospital-specific random effects from a normal distribution: $\beta_{0j} \sim N(0,\tau^{2} )$, where $\tau^{2}$ was determined so that the underlying random effects logistic regression model would have the desired ICC (or VPC), using the formula: ${\text{ICC}} = \frac{{\tau^{2} }}{{\tau^{2} + \pi^{2} /3}}$ [19]. These 50 hospital-specific random effects were then fixed for the remainder of the simulations in the given scenario.

We then simulated a baseline covariate for each subject from a standard normal distribution: $x_{ij} \sim N(0,1)$ for the *i*th patient at the *j*th hospital. Since the mean intercept and the fixed slope ($\beta_{0} ,\beta_{1} )$ are fixed within a given scenario, we computed the linear predictor for each subject: ${\text{LP}}_{ij} = \beta_{0} + \beta_{0j} + \beta_{1} x_{ij}$. Within each hospital, the predicted number of deaths was determined as: $\sum\limits_{i = 1}^{N} {\frac{{\exp (\beta_{0} + \beta_{0j} + \beta_{1} x_{ij} )}}{{1 + \exp (\beta_{0} + \beta_{0j} + \beta_{1} x_{ij} )}}}$, while the expected number of deaths was determined as $\sum\limits_{i = 1}^{N} {\frac{{\exp (\beta_{0} + \beta_{1} x_{ij} )}}{{1 + \exp (\beta_{0} + \beta_{1} x_{ij} )}}}$ (note that the latter sum differs from the former only by the exclusion of the cluster-specific random effect). Each hospital’s true predicted-to-expected ratio was computed as the ratio of these two quantities. These ratios are the true ratios and are fixed across simulation replicates. We will determine the empirical coverage rate of estimated 95% confidence intervals. The hospital-specific random effects, the subjects’ baseline covariates, and the true predicted-to-expected ratios are fixed within each scenario and do not change across the simulation replicates.

Within a given simulation replicate we generated an outcome for each subject using the true linear predictor: ${\text{LP}}_{ij} = \beta_{0} + \beta_{0j} + \beta_{1} x_{ij}$. From the true linear predictor, we determined $p_{ij} = \frac{{\exp ({\text{LP}}_{ij} )}}{{1 + \exp ({\text{LP}}_{ij} )}}$, the subject-specific probability of the occurrence of the outcome. We then generated a binary outcome using a Bernoulli distribution with this subject-specific probability. For each of the 18 scenarios we created 200 datasets using this process (so that each scenario involved 200 simulation replicates).

### Statistical analyses in the simulated samples

In each simulation replicate we conducted the following analyses: (i) we fit a random effects logistic regression model in which the binary outcome was regressed on the continuous baseline covariate. The model incorporated cluster-specific random effects. The predicted-to-expected ratio was computed for each of the 50 clusters, resulting in 50 cluster-specific predicted-to-expected ratios; (ii) we drew 1000 bootstrap samples from the simulated sample for the given simulation replicate; (iii) in each bootstrap sample we fit a random effects logistic regression model (using a procedure identical to that in step (i)) and computed the predicted-to-expected ratio for each of the 50 clusters (we thus had 1000 predicted-to-expected ratios for each of the 50 clusters); (iv) we constructed 95% confidence intervals for each hospital’s predicted-to-expected ratio. This was done using normal-theory bootstrap methods and percentile-based bootstrap methods. For the normal-theory bootstrap method, for each hospital, we computed the standard deviation of the estimated predicted-to-expected ratios across the 1000 bootstrap replicates. This quantity serves as an estimate of the standard error of the estimated predicted-to-expected ratio. A 95% confidence interval for each hospital’s predicted-to-expected ratio was then computed as the estimated predicted-to-expected ratio from the original simulated sample ± 1.96 × the bootstrap estimate of the standard error of the predicted-to-expected ratio. For the percentile-based bootstrap method, the end points of the 95% confidence interval were the 2.5^th^ and 97.5^th^ percentiles of the predicted-to-expected ratios across the 1000 bootstrap samples.

We then conducted the following analyses across the 200 simulation replicates. First, for each of the 50 clusters we determined the ratio of the mean bootstrap estimate of the standard error of the predicted-to-expected ratio across the 200 simulation replicates to the standard deviation of the estimated predicted-to-expected ratio across the 200 simulation replicates. If this ratio is equal to one, then the bootstrap estimate of the standard error of the predicted-to-expected ratio is correctly approximating the standard deviation of the sampling distribution of the predicted-to-expected ratio. Thus, we obtained 50 such ratios, one for each of the 50 clusters. Second, for each of the two types of bootstrap confidence intervals (normal-theory based or percentile-based), we determined the proportion of estimated 95% confidence intervals that contained the true value of the predicted-to-expected ratio for that cluster. If the estimated confidence intervals had the correct coverage rates, we would expect that 95% of the constructed confidence intervals contain the true value of the predicted-to-expected ratio for that hospital.

We examined four different bootstrap procedures. First, we used the standard bootstrap in which subjects were sampled with replacement and the multilevel structure of the sample was not accounted for. We will refer to this as the naïve bootstrap. Second, we used a within-cluster bootstrap, in which a bootstrap sample of subjects is selected from within each cluster. Third, we used the parametric bootstrap procedure described above (this procedure was included despite our hypothesis that it would not perform well). Fourth, we used the bootstrap procedure for making inferences about cluster-specific random effects that was described above.

The simulations were conducted using the R statistical programming language (version 3.6.3). Random effects logistic regression models were fit using the glmer function in the lme4 package (version 4_1.1–21).

## Monte Carlo simulations: results

We report our results separately for each of the four bootstrap procedures.

### Naïve bootstrap

**Fig. 1:**
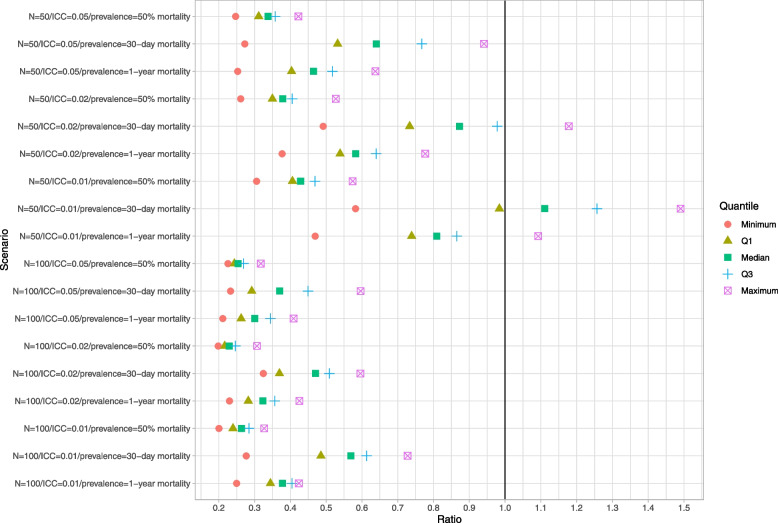
Ratio of mean estimated standard error to standard deviation of sampling distribution (naive bootstrap)

**Fig. 2:**
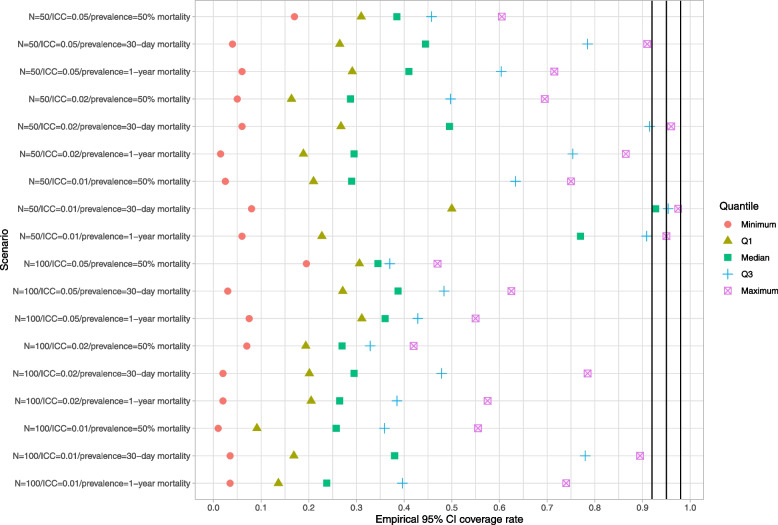
Empirical coverage rates of 95% bootstrap CIs (normal-theory method) (naive bootstrap)

**Fig. 3:**
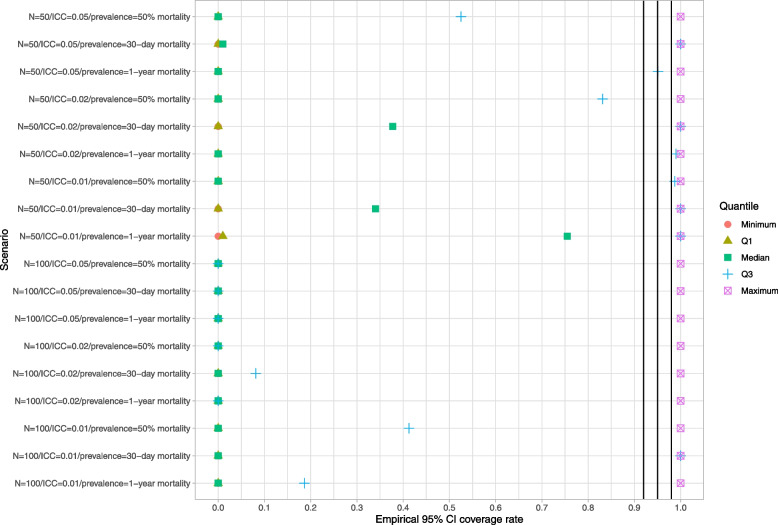
Empirical coverage rates of 95% bootstrap CIs (percentile method) (naive bootstrap)

Results for the naïve bootstrap are reported in Fig. 1 (ratio of mean estimated standard error to empirical standard error), Fig. 2 (coverage of 95% confidence intervals using the bootstrap with normal-theory methods), and Fig. 3 (coverage of 95% confidence intervals using bootstrap percentile intervals). Each figure is a dot chart, with one horizontal line for each of the 18 scenarios. On each horizontal line there are 5 dots, representing the minimum, 25^th^ percentile, median, 75^th^ percentile, and maximum quantity (ratio or empirical coverage rate) across the 50 clusters. On Fig. 1 we have superimposed a vertical line denoting a ratio of 1. On Figs. 2 and 3 we have superimposed verticals denoting the advertised coverage rate of 0.95. On the latter two figures we have also superimposed vertical lines denoting coverage rates of 0.92 and 0.98. Due to our use of 200 simulation replicates, empirical coverage rates that are less than 0.92 or greater than 0.98 are significantly different from the advertised rate of 0.95 using a standard normal-theory test.

We provide a guide to interpreting Fig. 1 (all subsequent figures have a similar interpretation). The top horizontal line denotes the scenario with 50 subjects per cluster, an ICC of 0.05, and an outcome prevalence of approximately 50%. Note that in the simulations we estimated 50 cluster-specific ratios of mean estimated standard error to empirical standard error (one ratio for each cluster). Across the 50 clusters, the lowest ratio of the mean estimated standard error to the empirical standard error was 0.25. Across the 50 clusters, the 25^th^ percentile of the ratio of the mean estimated standard error to the empirical standard error was 0.31. Across the 50 clusters, the median ratio of the mean estimated standard error to the empirical standard error was 0.34. Across the 50 clusters, the 75^th^ percentile of the ratio of the mean estimated standard error to the empirical standard error was 0.36. Finally, across the 50 clusters, the largest ratio of the mean estimated standard error to the empirical standard error was 0.42. These five quantities are represented by five different plotting symbols along the horizontal line. Note that all five quantities are to the left of the vertical line denoting a ratio of one. Thus, for none of the 50 clusters was the mean estimated standard error an accurate estimate of the empirical standard error.

In examining Fig. 1, we observe that across most of the 18 scenarios, the bootstrap estimate of the standard error of the predicted-to-expected ratio underestimated the standard deviation of the sampling distribution of the predicted-to-expected ratio across the 50 clusters. In general, the naïve bootstrap provided a poor estimate of the standard error of the predicted-to-expected ratio.

In examining Figs. 2 and 3, we observe that both bootstrap methods for constructing confidence intervals tended to result in 95% confidence intervals with lower then advertised coverage rates. The performance of the bootstrap percentile interval approach was particularly poor, with at least half the clusters having confidence intervals whose empirical coverage rates were zero in 15 of the 18 scenarios.

These analyses demonstrate that the use of the naïve bootstrap results in inaccurate estimates of standard error and confidence intervals with lower than advertised coverage rates.

### Within-cluster bootstrap

**Fig. 4:**
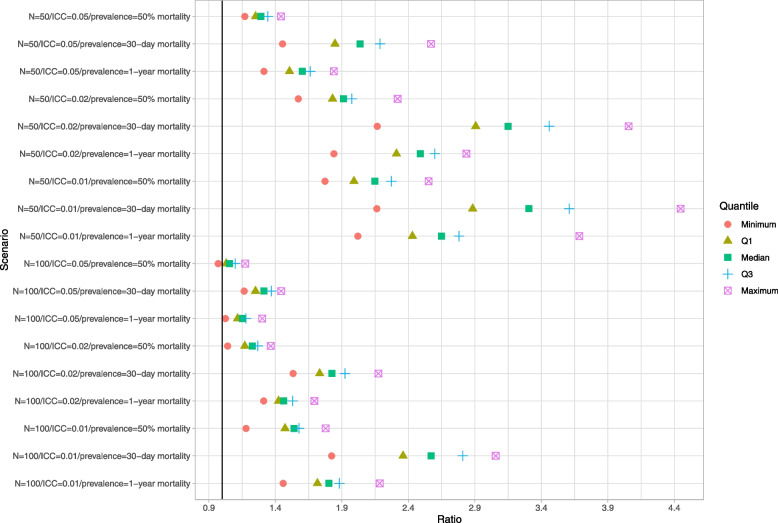
Ratio of mean estimated standard error to standard deviation of sampling distribution (within-cluster bootstrap)

**Fig. 5:**
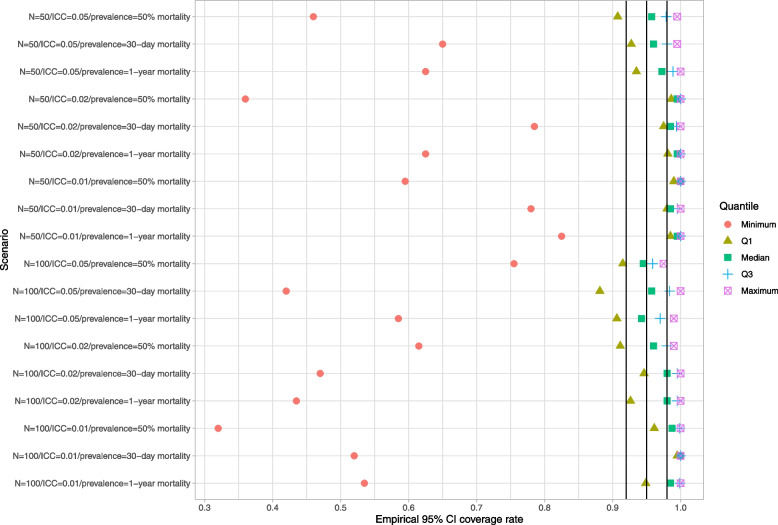
Empirical coverage rates of 95% bootstrap CIs (normal-theory method) (within-cluster bootstrap)

**Fig. 6:**
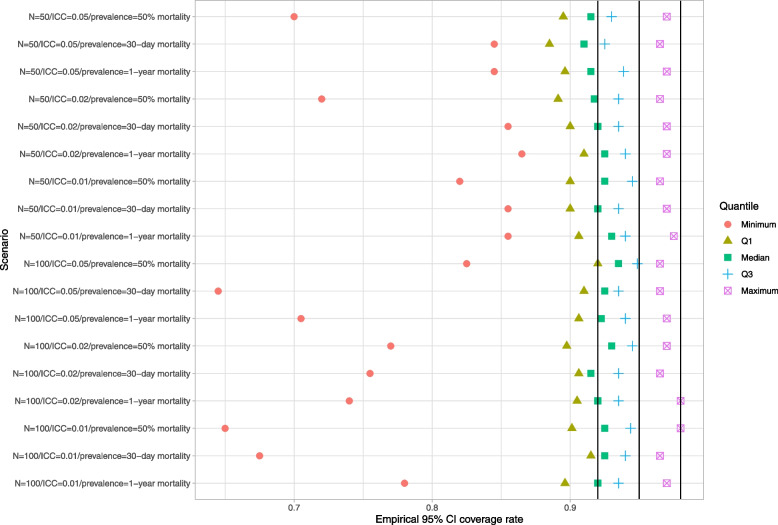
Empirical coverage rates of 95% bootstrap CIs (percentile method) (within-cluster bootstrap)

Results are reported in Figs. 4, 5 and 6. These figures have a structure similar to those of Figs. 1,2 and 3. The use of the within-cluster bootstrap substantially over-estimated the standard deviation of the sampling distribution of the predicted-to-expected ratios. The magnitude of over-estimation tended to be greater with 50 subjects per cluster than with 100 subjects per cluster. Empirical coverage rates of 95% confidence intervals, while still suboptimal, tended to be better than with the naïve bootstrap. For example, with normal-theory confidence intervals, there were clusters for which the empirical coverage rate was less than 0.85 across all 18 scenarios (and below 0.40 in some scenarios). However, in the majority of scenarios, at least 75% of the clusters had confidence intervals whose coverage rate was at least 92%. While the use of bootstrap percentile intervals tended to not be as good as the use of normal-theory methods, it was substantially better than what was observed for the bootstrap percentile intervals with the naïve bootstrap.

### Parametric bootstrap

**Fig. 7:**
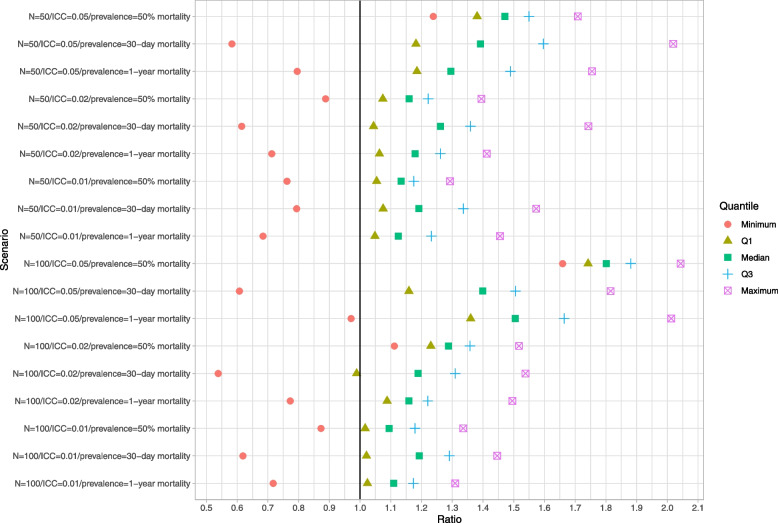
Ratio of mean estimated standard error to standard deviation of sampling distribution (parametric BS)

**Fig. 8:**
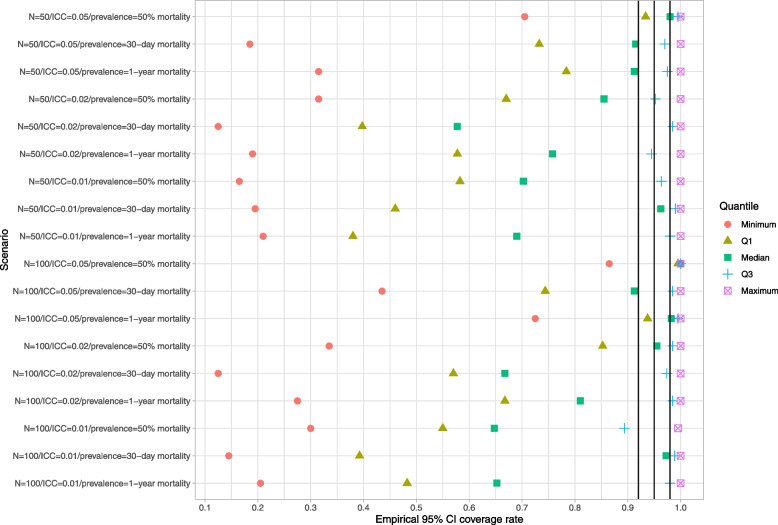
Empirical coverage rates of 95% bootstrap CIs (normal-theory method) (parametric BS)

**Fig. 9:**
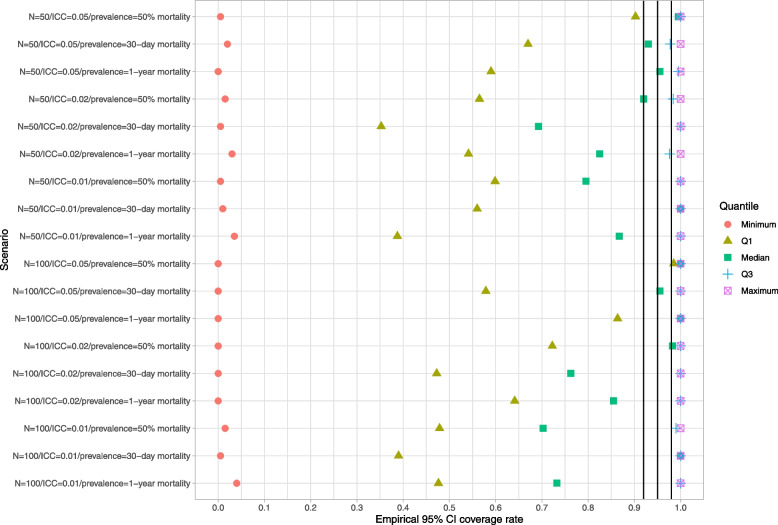
Empirical coverage rates of 95% bootstrap CIs (percentile method) (parametric BS)

Results for the parametric bootstrap are reported in Figs. 7, 8 and 9. These figures have a structure similar to those of Figs. 1, 2 and 3. The parametric bootstrap resulted in inaccurate estimates of the standard error of the cluster-specific predicted-to-expected ratios. Across the 18 scenarios, the use of the parametric bootstrap tended to over-estimate the standard deviation of the sampling distribution of the predicted-to-expected ratio. Both bootstrap-based methods for estimating confidence intervals tended to produce confidence intervals whose empirical coverage rates were significantly different than the advertised rate. In the majority of scenarios, at least half the clusters had estimated confidence intervals whose empirical coverage rate was less than 92% when using the normal-theory method. A similar finding was observed when bootstrap percentile intervals were used.

### Cluster-specific parametric bootstrap

**Fig. 10:**
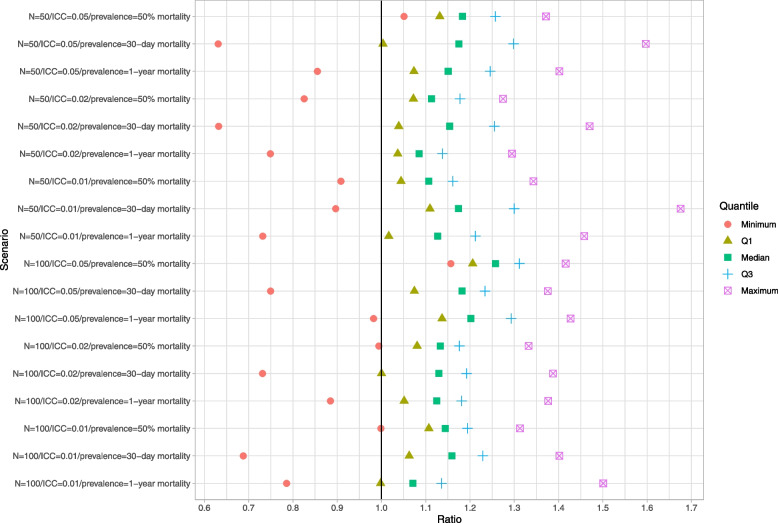
Ratio of mean estimated standard error to standard deviation of sampling distribution (cluster-specific parametric BS)

**Fig. 11:**
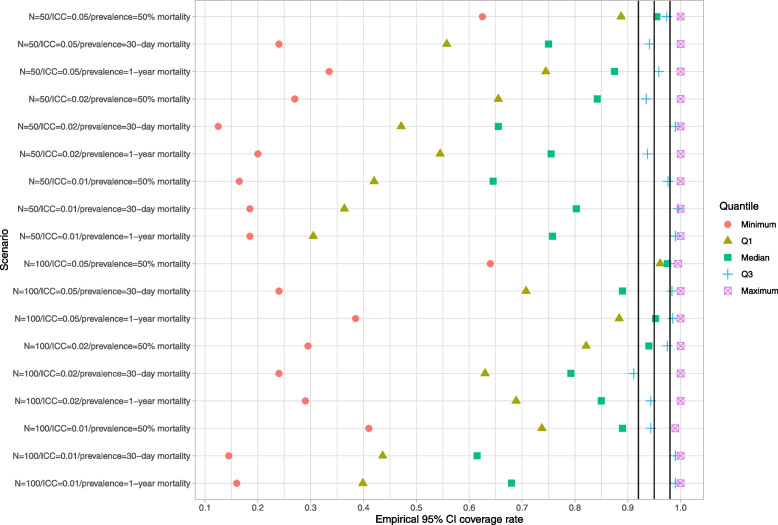
Empirical coverage rates of 95% bootstrap CIs (normal-theory method) (cluster-specific parametric BS)

**Fig. 12:**
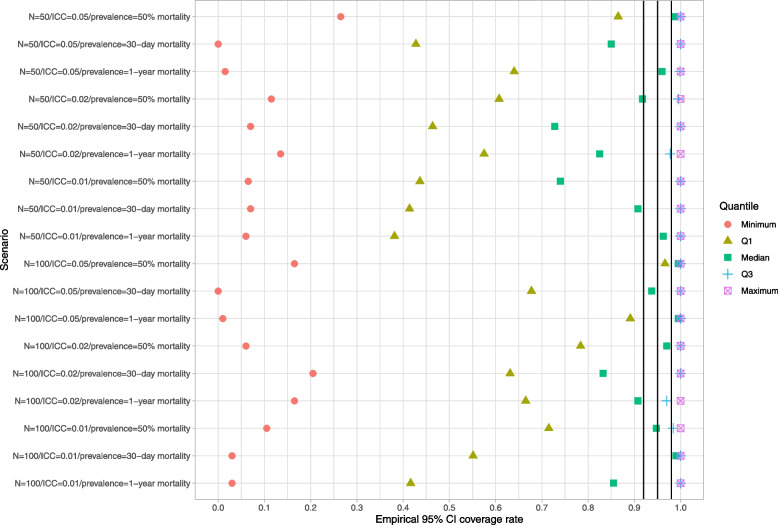
Empirical coverage rates of 95% bootstrap CIs (percentile method) (cluster-specific parametric BS)

Results are reported in Figs. 10, 11 and 12. These figures have a structure similar to those of Figs. 1, 2 and 3. In general, this bootstrap procedure resulted in estimated standard errors for the predicted-to-expected ratios that were larger than the standard deviation of the sampling distribution of the predicted-to-expected ratios. For each of the 18 scenarios, half of the clusters had a ratio of estimated standard error to standard deviation that exceeded about 1.15. Estimated confidence intervals (obtained using both normal-theory methods and using bootstrap percentile intervals) tended to have empirical coverage rates that were substantially lower than advertised.

## Case study

We provide a case study illustrating the application of the four bootstrap procedures to a sample of 19,559 patients hospitalized with a diagnosis of AMI at 157 hospitals.

### Methods

We used the OMID dataset that was described above. The outcome was death within 30 days of hospital admission. We used the 11 variables in the Ontario AMI Mortality Prediction Model (described above) for risk-adjustment. We regressed the binary outcome on these 11 variables using a random effects logistic regression model that incorporated hospital-specific random effects. The fitted model was ${\text{logit}}(\Pr (Y_{ij} = 1)) = \beta_{0} + \beta_{0j} + \beta_{1} X_{1ij} + \cdots + \beta_{11} X_{11ij}$, where $Y_{ij}$ denotes the binary outcome for the *i*th patient at the *j*th hospital, and $X_{1ij}$ through $X_{11ij}$ denote the 11 variables used for risk adjustment. We assume that $\beta_{0j} \sim N(0,\tau^{2} )$, where $\beta_{0j}$ denotes the random effect for the *j*th hospital.

The predicted-to-expected ratio was computed for each hospital. Each of the four bootstrap procedures was used to compute 95% confidence intervals around each hospital’s predicted-to-expected ratio. For each bootstrap procedure we constructed two confidence intervals: one using normal-theory methods and one using bootstrap percentile intervals.

For comparative purposes we also fit the random effects model within a Bayesian framework using Markov Chain Monte Carlo (MCMC) methods [20]. Diffuse non-informative priors were assumed for all model parameters: $\beta_{k} \sim N(0,\sigma^{2} = 10,000),{\text{ for }}k = 0,1,...,11$ and $\tau^{2} \sim \Gamma^{ - 1} ({\text{shape = 0}}{\text{.01, scale = 0}}{.01)}$, where $\Gamma^{ - 1}$ denotes the inverse Gamma distribution. Bayesian 95% credible intervals were computed for each hospital’s predicted-to-expected ratio using MCMC methods.

### Results

**Fig. 13:**
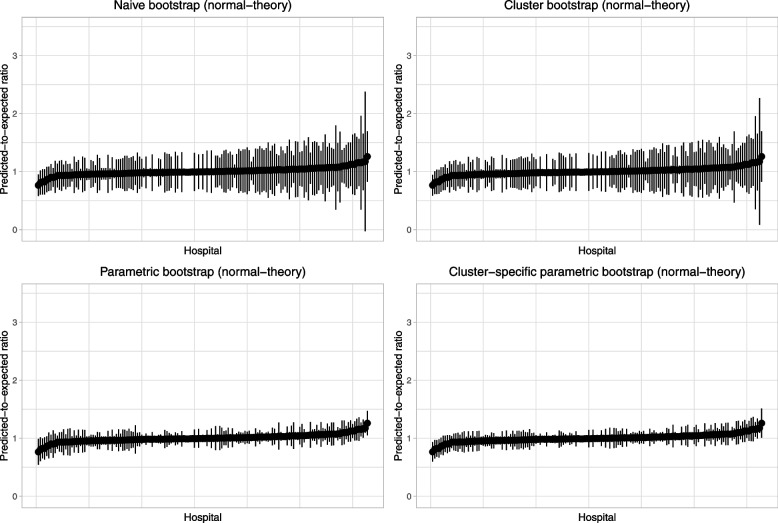
Caterpillar plots for frequentist analyses with bootstrap (normal-theory method)

**Fig. 14:**
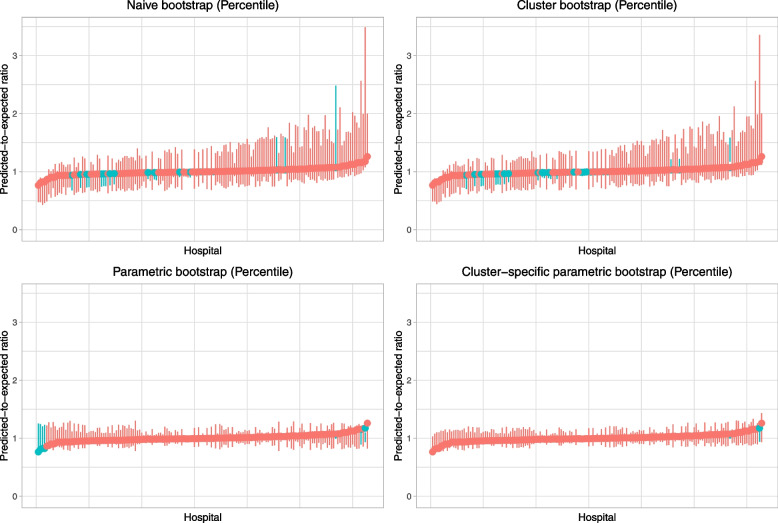
Caterpillar plots for frequentist analyses with bootstrap (percentile intervals)

Caterpillar plots illustrating each hospital’s predicted-to-expected ratio and its estimated 95% confidence interval are reported in Fig. 13 (normal-theory bootstrap confidence intervals) and Fig. 14 (bootstrap percentile intervals). Each figure has four panels, one for each of the four bootstrap procedures. All eight panels use the same scale for the vertical axis (the predicted-to-expected ratio). When using bootstrap percentile intervals, some of the estimated 95% confidence intervals did not contain the estimated predicted-to-expected ratio. Confidence intervals in Fig. 14 are reported using two colours (red: confidence interval contains the estimated predicted-to-expected ratio; blue: confidence interval does not contain the estimated predicted-to-expected ratio). The number of hospitals with problematic bootstrap percentile intervals were 19 (naïve bootstrap), 28 (cluster bootstrap), 7 (parametric bootstrap), and 2 (cluster-specific parametric bootstrap). In examining Figs. 13 and 14, one notes wide variation in the caterpillar plot across the eight panels. When using bootstrap percentile methods, one observes that the estimated confidence intervals were often substantially asymmetric (i.e., the point estimate did not lay in the centre of the interval). Furthermore, the widths of the intervals varied across bootstrap procedures.

**Fig. 15:**
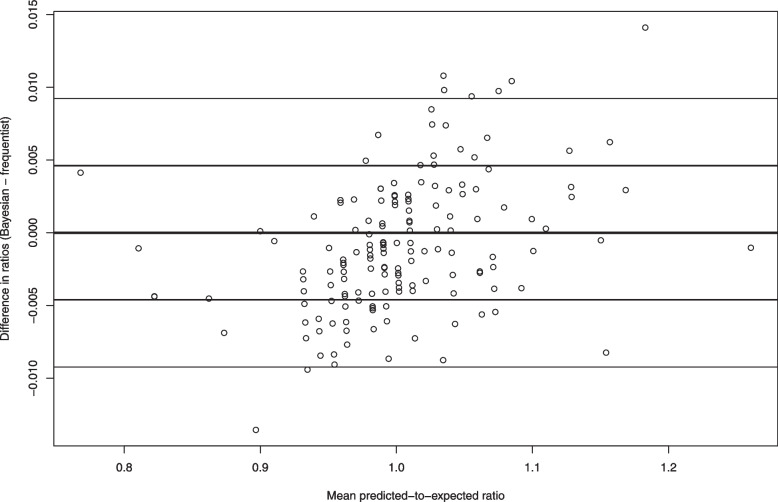
Bland−Altman plot comparing Bayesian and frequentist predicted-to-expected ratios

Figure 15 contains a Bland–Altman plot comparing the agreement between the frequentist and Bayesian predicted-to-expected ratios. On this figure we have superimposed horizontal lines denoting ± 1 standard deviation and ± 2 standard deviations from zero (no difference). We see that, for the large majority of hospitals, the two predicted-to-expected ratios were within 0.01 of each other.

**Fig. 16:**
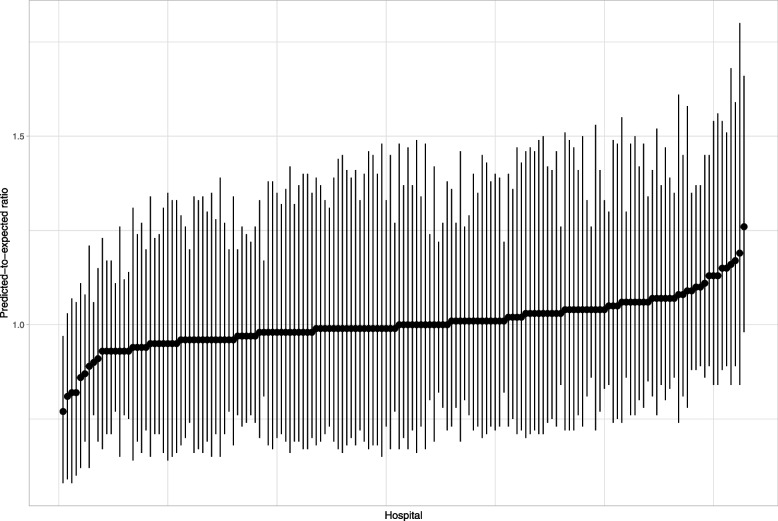
Caterpillar plot for 30-day predicted-expected ratio for Bayesian analysis

Figure 16 reports the caterpillar plot resulting from the Bayesian analysis. Only one hospital had a 95% credible interval that excluded unity. We note that the credible intervals display greater symmetry than did the bootstrap percentile intervals in Fig. 14. The Bayesian credible intervals displayed less variability in width than did the bootstrap confidence intervals. The ratio of the longest to short width for the Bayesian intervals was 3.2, while this ratio ranged from 12.8 to 222.9 across the eight combinations of bootstrap procedures and methods for constructing confidence intervals.

## Discussion

We examined the performance of four bootstrap procedures for estimating confidence intervals for provider-specific predicted-to-expected ratios. We found that all four bootstrap procedures had suboptimal performance.

The primary limitation of the current study was its reliance on Monte Carlo simulations. Such simulations were necessary since we were examining the performance of resampling-based procedures, for which analytic derivations are not feasible. Due to our use of simulations, we could only examine a limited number of scenarios due to the time-intensive nature of these simulations. Despite considering a limited number of scenarios, the performance of the different bootstrap procedures was consistently poor across these scenarios, indicating that, in general, these bootstrap procedures should not be used for estimating confidence intervals for predicted-to-expected ratios. A second limitation was that the simulations only used 200 iterations per scenario. The rationale for this decision was the computational intensity of simulations of bootstrapping of random effects models. For example, with 200 iterations per scenario, the simulations for the four bootstrap procedures required approximately 23, 27, 29, and 30 days of CPU-time for the 18 scenarios (for a total of approximately 109 days of CPU-time). Increasing the number of simulation replicates to 1000 would have been prohibitive in terms of computation time. With the use of 200 simulation replicates, empirical coverage rates that are less than 0.92 or greater than 0.98 are significantly different from the advertised rate of 0.95 using a standard normal-theory test.

In the current study we focused on frequentist estimation of the random effects model used for computing the predicted-to-expected ratios. An alternative approach, as illustrated in the case study, would be to use Bayesian methods to estimate the posterior distribution of the model parameters and the resultant predicted-to-expected ratios. Different authors have suggested that Bayesian methods be used for provider profiling [21, 22], while several studies have evaluated the performance of Bayesian methods for provider profiling [23–27]. There are several advantages to the use of Bayesian methods. First, when using MCMC methods to estimate the posterior distribution of the model parameters, one can directly compute the predicted-to-expected ratios within each iteration of the MCMC process. This allows for directly computing credible intervals (the Bayesian analogue to confidence intervals) for the predicted-to-expected ratios. Second, rather than simply report the predicted-to-expected ratios and their associated credible intervals, Bayesian methods allow for the reporting of other policy-relevant metrics, such as the probability that the predicted-to-expected ratio exceeds a predetermined policy-relevant threshold (e.g., the probability that the predicted-to-expected ratio exceeds 1.25). Given the absence of a closed-form expression for the standard error of the estimate of the predicted-to-expected ratio and the observed failure of different bootstrap procedures, we suggest that authors who want to use predicted-to-expected ratios work within a Bayesian framework.

Direction for future research includes developing closed-form expressions for the standard error of the predicted-to-expected ratios or of developing bootstrap procedures that are appropriate for use with these measures of provider performance.

## Conclusions

Four bootstrap procedures were observed to result in inaccurate estimates of the standard errors of healthcare providers’ predicted-to-expected ratios and in confidence intervals that did not have the advertised coverage rates. We recommend that Bayesian methods be used for analyses involving predicted-to-expected ratios.

## Data Availability

The dataset from this study is held securely in coded form at ICES. While legal data sharing agreements between ICES and data providers (e.g., healthcare organizations and government) prohibit ICES from making the dataset publicly available, access may be granted to those who meet pre-specified criteria for confidential access, available at www.ices.on.ca/DAS (email: das@ices.on.ca).

## Abbreviations

AMI: Acute myocardial infarction
CABG: Coronary artery bypass graft
CPU: Central processing unit
ICC: Intraclass correlation coefficient
OMID: Ontario Myocardial Infarction Database
MCMC: Markov Chain Monte Carlo
VPC: Variance partition coefficient
